# Mandibular molar uprighting using orthodontic miniscrew implants: a systematic review

**DOI:** 10.1186/s40510-017-0200-2

**Published:** 2018-01-08

**Authors:** Panagiota Magkavali-Trikka, Georgios Emmanouilidis, Moschos A. Papadopoulos

**Affiliations:** 1 Hamdan Bin Mohammed College of Dental Medicine, Mohammed Bin Rashid University of Medicine and Health Sciences, Dubai, United Arab Emirates; 2 Department of Orthodontics, School of Health Sciences, Faculty of Dentistry, Aristotle University of Thessaloniki, Thessaloniki, Greece

## Abstract

The purpose of this systematic review was to identify studies and present the use of miniscrew implants (MIs) as an alternative treatment to mandibular molar uprighting. An electronic search and handsearching were conducted by two independent reviewers to identify relevant articles, published up to January 27, 2017. In order to methodologically assess the eligible studies, a pilot checklist consisting of 22 items was also implemented. After exclusion of all the irrelevant papers, only 17 studies were included, presenting 27 cases of mandibular molar uprighting in all planes using both direct and indirect force traction by MIs. Regarding the quality evaluation, the mean score of the included studies was 13.2, indicating a rather poor methodology implemented in the majority of the included cases. Due to many advantages, MIs provide a unique treatment alternative and constitute a reliable solution for treating tipped or impacted molars. Regarding the force application, a direct method is simpler, as it requires one MI and a single bracket or button, minimizing the patient’s discomfort and also reducing chair time compared to more complex indirect anchorage. It also eliminates the possibility of unwanted movement of the anchorage unit, which can occur even with indirect anchorage as a result of technical errors. However, direct anchorage has limitations in cases of lingually tipped or rotated molars because a single force may be insufficient to upright the tooth.

## Background

A tipped mandibular molar is a frequent situation among orthodontic patients, which usually occurs after premature loss of adjacent teeth leading to the inclination of the molars [1, 2]. Inadequate mandibular arch length, excessive teeth size, loss of the adjacent first molar, premature eruption of the mandibular third molar, and unusually mesial eruption pathway of the second molar can also cause its partial or total impaction with a reported incidence of 0.03–0.3% of the general population and 2–3% of orthodontic patients [3–5].

Tilted molars can cause numerous problems in a patient’s mouth, especially if a prosthetic rehabilitation is planned. According to Zachrisson, periodontal status can be aggravated, with signs of inflammation, angular bone loss, and an apparent pocket at the mesial surface of a tipped mandibular molar [6]. In excessive inclination, overeruption of the antagonist molar, premature contacts, and occlusal interferences impede prosthetic restoration [7]. However, molar uprighting into its correct position leads to the normalization of the functional and periodontal condition [7]. Finally, an impacted mandibular second molar can lead to caries, periodontal disease, or external root resorption of the adjacent first molar [4].

### Conventional methods for molar uprighting

Several orthodontic approaches are suggested for mandibular molar uprighting, such as Australian uprighting spring, cantilever spring, prefabricated Sander spring, helical uprighting spring, NiTi coil spring, push spring appliance, and traction from removable appliances are few of the currently available options [6, 8, 9]. Molar uprighting requires good anchorage control, and subsequently, a full-arch fixed appliance is necessary. Furthermore, ankylosed teeth, dental implants, and extraoral appliances could also be effective, enhancing anchorage, and protecting from undesirable tooth movements [8, 10].

Among others, the Uprighter Jet developed by Carano provides a complete control of molar uprighting, minimizing extrusion, requiring no brackets, and no special patient cooperation [1]. In another uprighting case of an impacted molar, in combination with rapid maxillary expansion (RME), vertical elastic forces were directed from a hook on the RME device to an orthodontic attachment bonded on the tooth to be uprighted. This method saves time, requires no additional anchorage preparation, and appears more physiologic as the force vector is in the direction of normal eruption path [9]. According to Pogrel [11], surgical uprighting of lower second molars is a quick procedure with minimal morbidity and long-term prognosis. Most of the uprighted teeth remained firm with excellent bone formation and periodontal status after 18 months of follow up [11].

However, conventional treatment methods for molar uprighting have some disadvantages, including extrusion of the target molar, unwanted reciprocal movement of the anchorage units, need for bulky appliances, and longer treatment time [1, 2, 12–15]. In order to minimize the first two side-effects, intra-arch stabilization is usually needed [2, 12, 13], which is undertaken through the use of osseointegrated implants. Yet, an osseointegrated dental implant is costly, needs sufficient bone space, limiting our choices, and is very difficult to be removed after the treatment. It also requires osseointegration before orthodontic force application, increasing the treatment time [5, 10, 12].

In addition, surgical uprighting should not be considered as a routine method due to the possible pulp necrosis, ankylosis, external root resorption, or even rupture during the procedure. After treatment, occlusal equilibration may be needed, and the post-surgical stability of the tooth may be questionable [4]. Further, the possibility of pulpal calcification and vitality loss is high [11].

### Use of orthodontic miniscrew implants for molar uprighting

The development of orthodontic miniscrew implants (MIs) provided solutions to most of the aforementioned problems. MIs are fabricated from pure titanium or titanium alloy with a diameter of 1–2 mm and length of 8–20 mm [16]. They remain stable during orthodontic treatment with minimum anchorage loss and are more effective than conventional anchorage means [17–19]. Their success rate ranges from 59 to 100% with an average of 86.5% [18–20].

Their attachment to the bone is mechanical with no intent to establish any form of osseointegration [21, 22]. Therefore, after the treatment, when they are no longer needed, they can be removed through a simple procedure, with negligible risks for the patient [23].

This new type of skeletal anchorage is simpler, smaller, less-invasive, and more economical than conventional osseointegrated implants [16]. Moreover, MIs do not require a long interval between placement and force application since loading can occur immediately after placement [10, 16, 24].

Their main advantage though is their ability to move specific teeth or even the entire quadrants directly, without involving other teeth or using inter-arch mechanics. Thus, they eliminate the reaction forces usually applied on the anchor teeth, leading to unwanted tooth movement and anchorage loss [5, 14, 15]. Patients are also more satisfied with the more invisible treatment compared to conventional methods [10].

MI anchorage is preferable to conventional mechanics when a third molar is in direct contact with the second molar root [4]. In some cases, it is better from a biomechanical perspective not to extract the third molar bud, since its extraction can change the center of resistance of the second molar and uprighting can be realized with distal crown tipping. This is undesirable when the second molar is planned to be uprighted mostly with mesial root tipping [24].

## Materials and methods

In order to find the appropriate articles to be included in this systematic review, Medline was electronically searched via PubMed on January 27, 2017, using the following search strategy:

(mini implants OR mini-implants OR screw implants OR miniscrew implants OR mini-screw implants OR mini screw implants OR microscrew implants OR micro-screw implants OR micro screw implants OR microimplants OR micro-implants OR micro implants) AND (orthodont*) AND ((molar) OR (“preprosthetic”) OR (upright) OR (upright*) OR (tipped) OR (tipping))

Random searching on Google Scholar and other databases was also held. All types of human studies including case reports were selected for inclusion. The reference lists of each article eligible for inclusion were also manually reviewed.

In order to methodologically assess the eligible case reports/series, we implemented a pilot checklist including several aspects. This checklist consists of 22 items in total, appropriately classified in seven domains: (1) patient, (2).treatment providers, (3) diagnostic evaluation, (4) appliance characteristics, (5) treatment procedure, (6) validity of results, and (7) conflict of interest. Each criterion could receive three possible ratings: 0 when the criterion was not fulfilled, 1 when no clear judgment could be reached, and 2 when the criterion was certainly fulfilled, resulting on a maximum score of 44 points per case. Finally, a judgment of the total quality of the respective case reports was made, based on the following interpretations: low quality when the total score was 0–11, lower medium quality when the total score was 12–22, upper medium quality when the total score was 23–33, and high quality when the total score was 34–44.

## Results

**Fig. 1:**
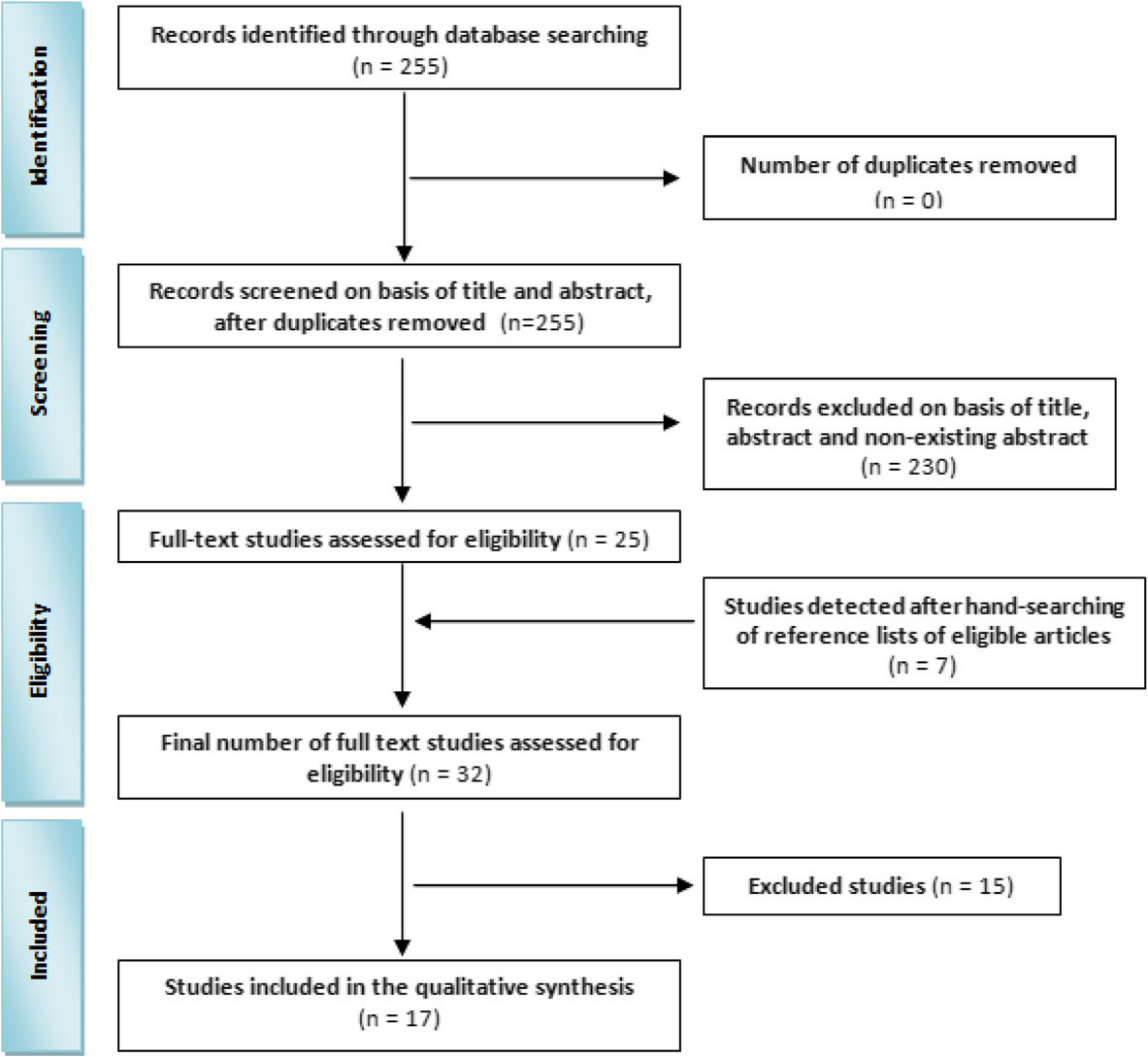
The PRISMA flow diagram for the selection of studies

Initially, 255 citations were found in total. Two hundred thirty citations were excluded as non-relevant, and 25 articles remained for further evaluation using their full-text. Through handsearching, 7 more articles were added. After excluding 15 non-relevant articles, only 17 papers remained for inclusion and their findings are reported in this systematic review (Fig. 1).

There are two methods of application of orthodontic forces when utilizing MIs: (a) using direct anchorage and (b) using indirect anchorage. Direct anchorage describes situations where the teeth desired to be moved are directly pulled or pushed towards or against the MIs. In contrast, indirect anchorage refers to the stabilization of certain teeth via a rigid connection with the MI and subsequent use of these stabilized anchors to move other teeth in the dental arch.

According to the current literature search, 27 cases were documented with both direct and indirect force traction by MIs, although most of them used the direct method (Table 1).

**Table 1:** Summary of cases included in this review

Author	Direct/indirect	Plane	Tooth	Insertion site	MI size	Quality
Sohn 2008 [10]	Direct	Transverse	Lingually tilted #47	Buccal alveolar bone of #47	1.4 × 6 mm	Lower medium
Park 2002 [12]	Direct	Sagittal and transverse	Mesially and lingually tipped #47 (crossbite)	Retromolar area (distobuccally, 10 mm from the distal surface of #47)	1.2 × 6 mm	Lower medium
	Direct	Sagittal	Mesially tipped lower second molar	Retromolar area	1.2 × 8 mm	Lower medium
Park 2004 [28]	Direct	Transverse	#37 and #27 in crossbite	1) Palatally between #26 and #27 2) Mandibular alveolar bone, buccal to #37 Both: 30–40° to the long axes of teeth	1.2 × 10 in the maxillary alveolar bone and 1.2 × 6 in the mandibular alveolar bone	Lower medium
Gracco 2007 [1]	Direct	Sagittal	Mesially inclined #37	MI was inserted manually through the buccal hole of the steel plate, which provided the most perpendicular positioning of the bayonet and tube	Length of 11 mm, head height of 2.25 mm, diameter of 0.8 mm at the tip and 1.25 mm at the head	Lower medium
Lee 2007 [4]	Direct	Sagittal	Mesially angulated 47 (locked)	Mesially, in the buccal alveolus between #46 and #45	1.8 × 7 mm	Lower medium
	Direct	Sagittal	Mesially impacted #37	Mesially, in the buccal alveolus between #36 and #35 (in the 2nd phase: between #35 and #34	No information	Lower medium
	Direct	Sagittal	Mesially impacted #47	Retromolar area	No information	Lower medium
Celebi 2011 [29]	Direct	Sagittal and transverse and vertical	#37 was mesially tipped and obliquely impacted under the distal bulge of #36	Buccal side between the first and second maxillary left molar’s roots	1.8 × 8 mm	Upper medium
Allgayer 2013 [5]	Direct	Sagittal	Mesially tipped lower second molars	Retromolar area	2 × 9 mm	Lower medium
Derton 2012 [15]	Direct	Sagittal	Mesially tipped first molar	Retromolar area	2 × 9 mm	Lower medium
	Direct	Sagittal	Third molar	Two mini-implants mesially to the molar	2 × 9 and 1.5 × 8 mm	Lower medium
Yun 2005 [13]	Indirect	Sagittal	Mesially tipped and partially impacted the second molar	Between the mandibular right second premolar and first molar	No information	Low
Lee 2009 [27]	Direct	Sagittal	Second mandibular molar	Between the right canine and the 1st premolar	No information	Upper medium
Nienkemper 2013 [26]	Direct	Sagittal	Mesially tipped #37	Perpendicular to the edentulous alveolar ridge	2 × 11 mm	Lower medium
	Direct	Sagittal	Mesially tipped #47	Perpendicular to the edentulous alveolar ridge	2 × 11 mm	Lower medium
Giancotti 2004 [3]	Direct	Sagittal	Deeply impacted #37 (with overerupted #38)	Retromolar area	2.3 × 7 mm	Low
Sivolella 2012^*^ [23]	Direct	Sagittal	Mesially impacted #47	Retromolar area	2 × 12 mm	Lower medium
Musilli 2010 [2]	Direct	Sagittal	Mesially tipped #37	Retromolar area	No information	Low
	Direct	Sagittal	Mesially tipped #37	Retromolar area	No information	Low
	Indirect	Sagittal	Mesially tipped#37 and #46	Mesial to the molars (it is not mentioned if they were placed perpendicular to the alveolar ridge or obliquely and it is not clear from the pictures)	No information	Low
Greco 2012 [25]	Direct	Sagittal	Mesially inclined #37	Retromolar area	1.3 × 12 mm	Lower medium
Melo 2013 [31]	65 MIs direct 116 MIs indirect			Vertically positioned on the alveolar crest or perpendicular to the buccal face of the alveolar bone	7, 9, or 11 mm in length and 1.3 or 1.6 mm in diameter	
Padmaprabha 2015 [30]	Direct	Transverse and vertical	Lingually tipped and supraerupted #46	In the interradicular region of #46	1.2 × 6 mm	Upper medium

^*^Five cases in total. Only one described. The rest are presented in the table

As far as the quality evaluation is concerned, the mean score of the included studies was 13.2, indicating a rather poor methodology implemented in the majority of the included cases. In detail, 9 cases were judged with low quality, 15 with lower medium quality, 3 with upper medium quality while none of the investigated case reports was found to present high quality (Table 2).

**Table 2:** Quality assessment of the included case reports-series using the pilot assessment scale

A/A	Cases	Score	Rating
1	Sohn 2008	14	lower medium quality
2	Park 2002 (case 1)	17	lower medium quality
3	Park 2002 (case 2)	12	lower medium quality
4	Park 2004	14	lower medium quality
5	Gracco 2007	19	lower medium quality
6	Lee 2007 (case 1)	15	lower medium quality
7	Lee 2007 (case 2)	19	lower medium quality
8	Lee 2007 (case 3)	15	lower medium quality
9	Celebi 2011	23	upper medium quality
10	Allgayer 2013	21	lower medium quality
11	Derton 2012 (Case 1)	15	lower medium quality
12	Derton 2012 (Case 2)	15	lower medium quality
13	Yun 2005	10	low quality
14	Lee 2009	25	upper medium quality
15	Nienkemper 2013 (Case 1)	12	lower medium quality
16	Nienkemper 2013 (Case 2)	13	lower medium quality
17	Giancotti 2004	8	low quality
18	Sivolella 2012 (Case 1)	14	lower medium quality
19	Sivolella 2012 (Case 2)	3	low quality
20	Sivolella 2012 (Case 3)	3	low quality
21	Sivolella 2012 (Case 4)	3	low quality
22	Sivolella 2012 (Case 5)	3	low quality
23	Musilli 2010 (case 1)	8	low quality
24	Musilli 2010 (case 2)	6	low quality
25	Musilli 2010 (case 3)	8	low quality
26	Greco 2012	14	lower medium quality
27	Padmaprabha 2015	28	upper medium quality

Rating was done according to the following: low quality when the total score was 0–11, lower medium quality for scores 12–22, upper medium quality for scores 23–33 and high quality for scores 34–44

### Molar uprighting using MIs with direct anchorage

Molar uprighting using MIs with direct anchorage was the sole or partial subject of the 15 included papers. One of these 15 papers [2] describes two cases treated with direct use of MIs and one case with indirect use, and this is why it will be discussed again in the following paragraph.

**Fig. 2:**
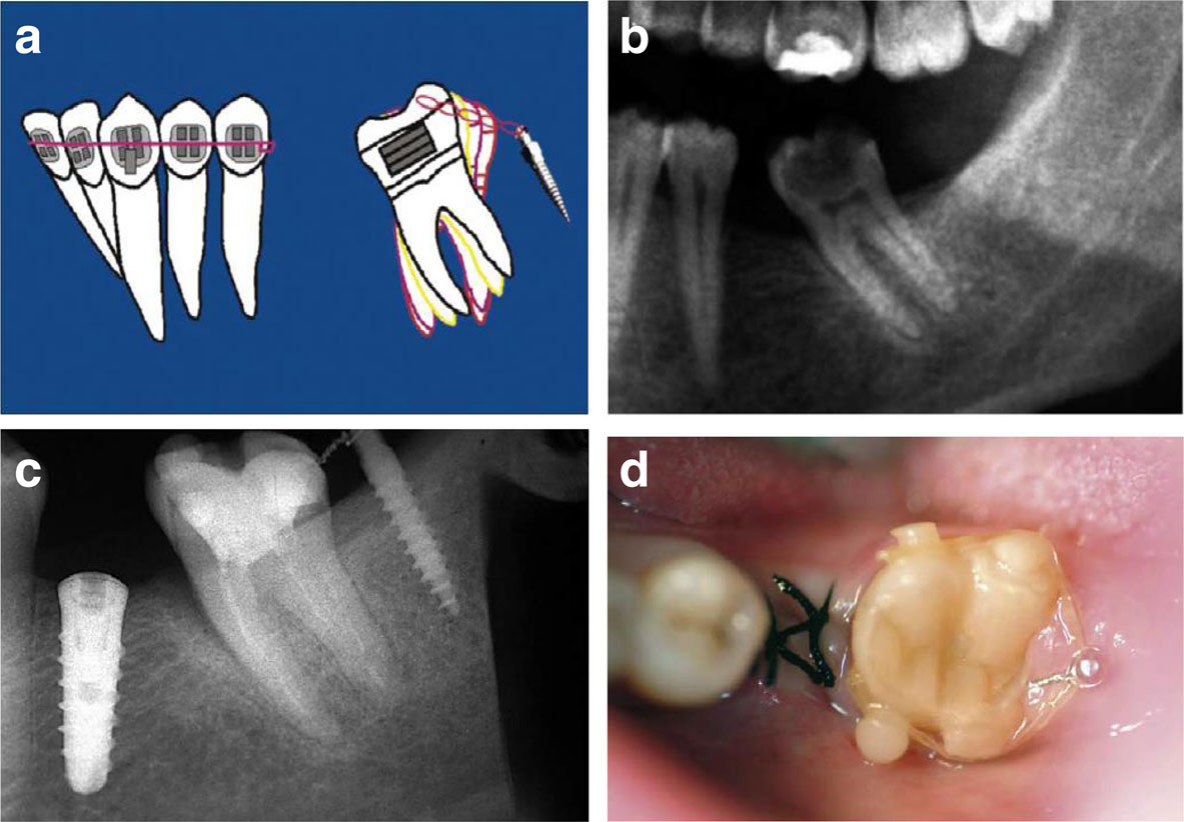
Uprighting of #37 with a miniscrew implant and elastomeric chain. **a** A miniscrew positioned in the retromolar area with an elastomeric chain between the screw and the molar; uncontrolled tipping. **b** Initial radiograph at the area of #37. **c** Final radiograph after the uprighting of #37, with the miniscrew placed distally and an implant in the site of #36. **d** Occlusal view of uprighted #37. (From Musilli et al. [2], with kind permission of Progress in Orthodontics)

**Fig. 3:**
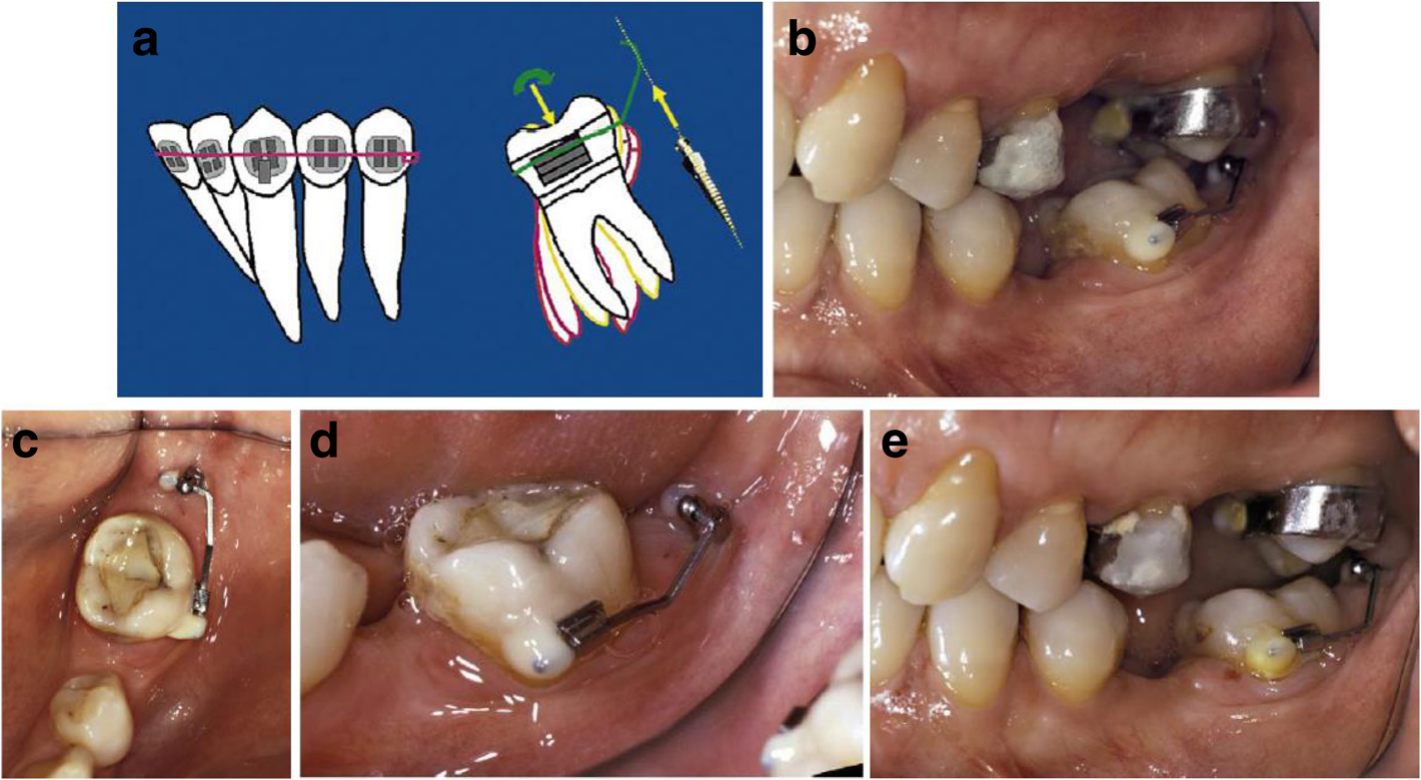
Uprighting of #37 with a miniscrew implant and a cantilever. **a** A miniscrew is placed in the retromolar area, and a small cantilever of beta-titanium wire is inserted in a small buccal tube on the labial surface of #37. The yellow and green arrows represent the force system on both the tooth and the TAD. **b** Uprighting of the molar with a cantilever connected from the tooth to the screw distal to the molar. **c** Occlusal view of #37 at the beginning of uprighting. **d** Lateral view of #37 at the beginning stage. **e** Lateral view of #37 at the end of uprighting. (From Musilli et al. [2], with kind permission of Progress in Orthodontics)

**Fig. 4:**
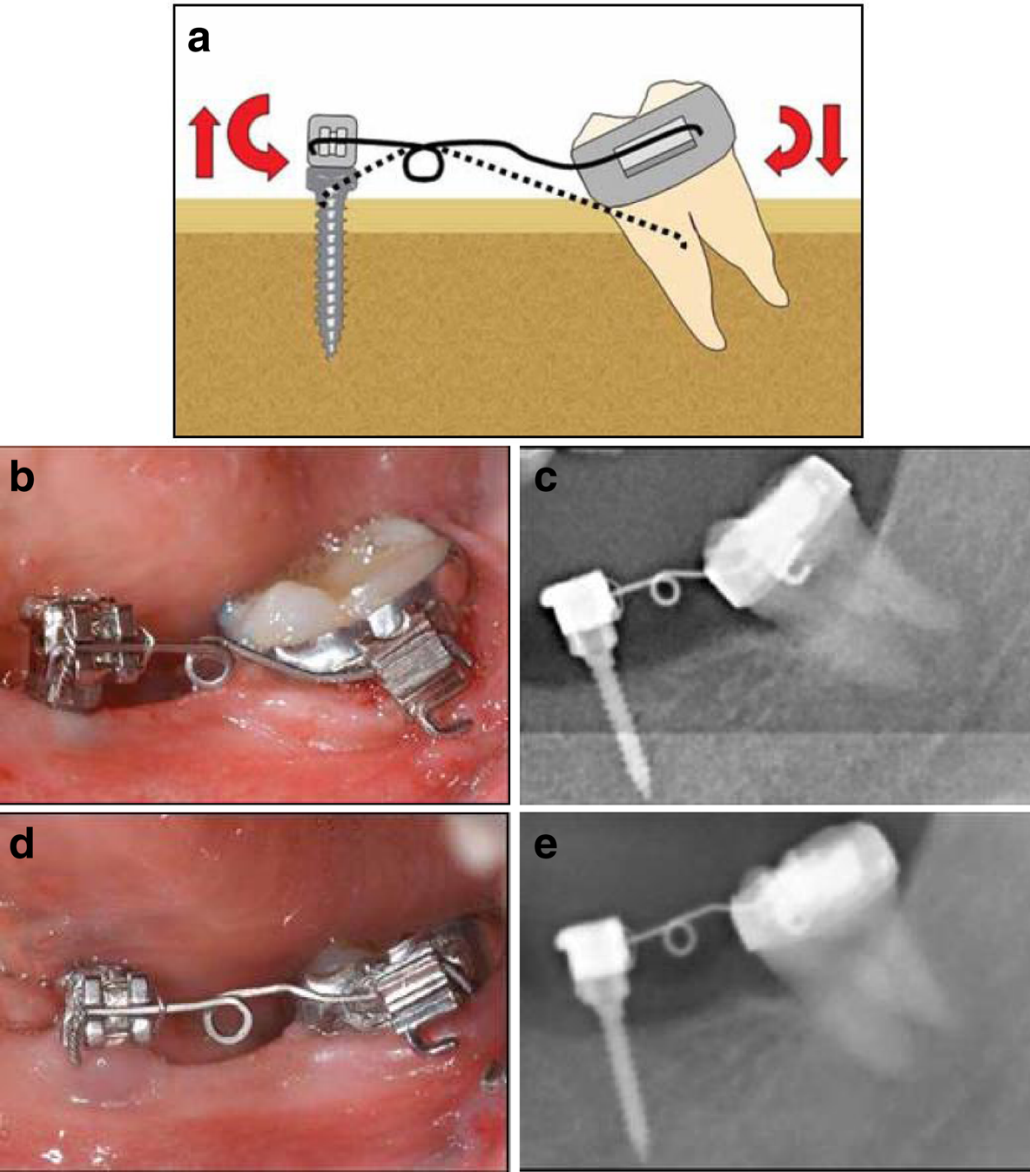
Preprosthetic uprighting of #37 with a miniscrew implant and an uprighting spring. **a** Activation of the uprighting spring with a mesial eccentric V-bend. **b**, **c** A mini-implant with an incorporated bracket abutment placed in the alveolar ridge as an attachment of the uprighting spring. **d**, **e** After 5 months of molar-uprighting treatment, no extrusion has taken place. (From Nienkemper et al. [26], with kind permission of Journal of Clinical Orthodontics)

**Fig. 5:**
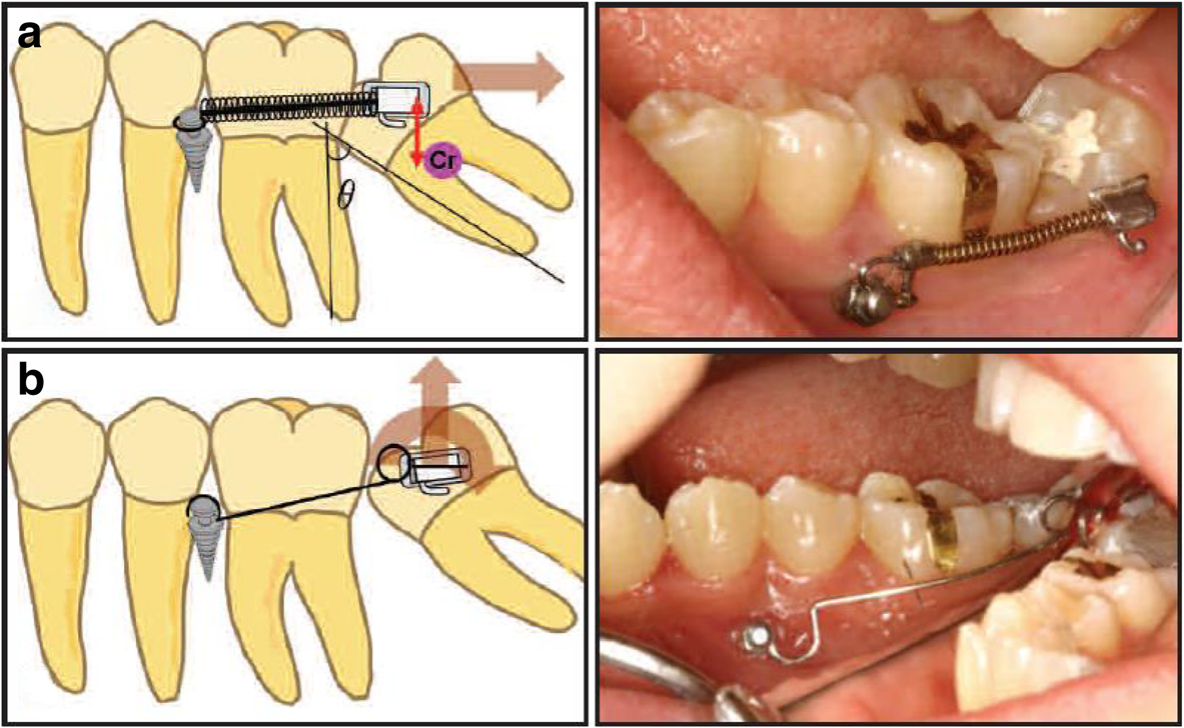
Uprighting of a mesially impacted #37 with miniscrew anchorage, an open coil spring and a stainless steel uprighting spring. **a** Step 1: Unlocking of the second molar with distalizing force from .016 in. stainless steel wire and open coil spring. **b** Step 2: Uprighting of the second molar with tip back moment from .016 × .022 in. stainless steel wire spring (from Lee et al. [4], with kind permission of Journal of Clinical Orthodontics)

Regarding the use of direct application of forces on the MIs for the correction of molars on the *sagittal plane*, cases of uprighting all three mandibular molars were found, which were initially either mesially tilted or impacted. In order to treat these cases, several options regarding the insertion sites of the MIs were used: (a) in the retromolar area [2–5, 12, 15, 23, 25] (Figs. 2 and 3), (b) vertically in the alveolar ridge of a mesial edentulous molar site [2, 26] (Fig 4), or (c) mesial to the mandibular molar and between the roots of the adjacent teeth [4, 15, 27] (Fig 5).

**Fig. 6:**
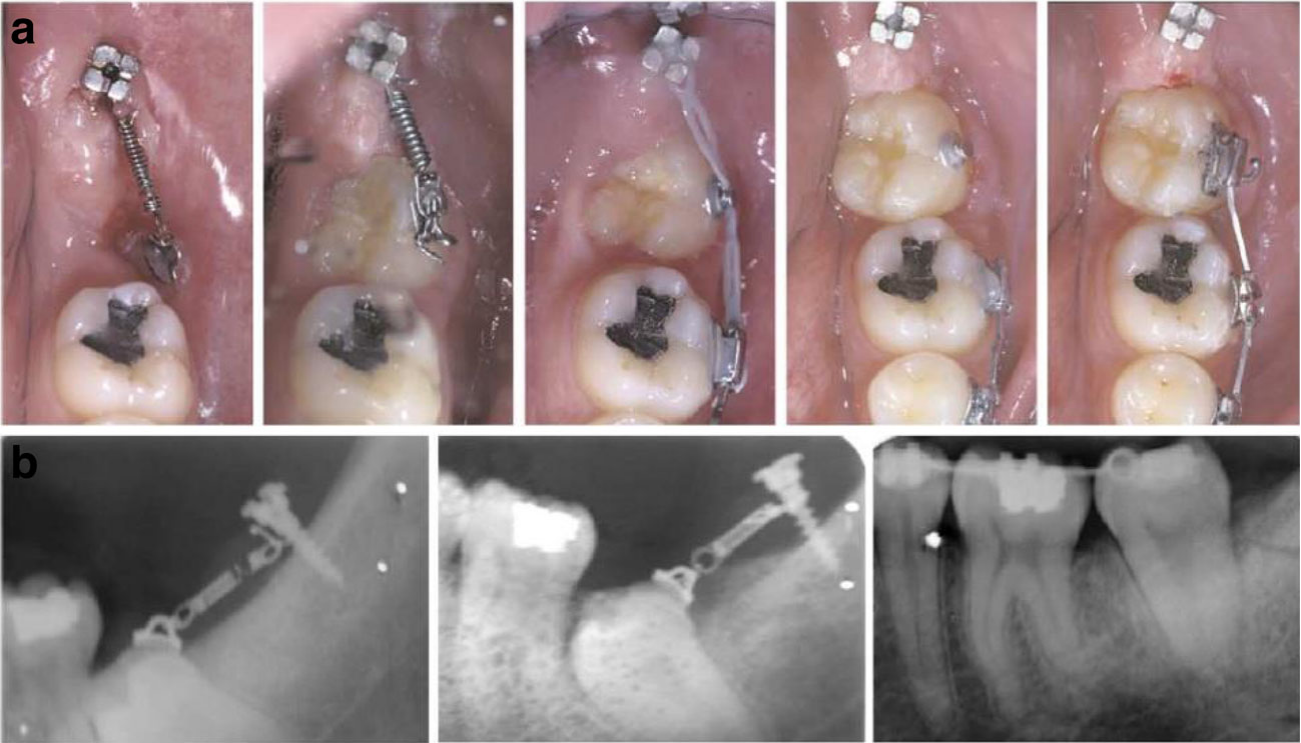
Uprighting of an impacted #37 with miniscrew implant and a closed coil spring tied to an orthodontic bracket. **a** A 7-mm titanium miniscrew inserted in the retromolar area and loaded with 50-g force on nickel-titanium closed coil spring. After the extrusion of the crown, the tooth was uprighted with fixed sectional appliance. **b** Progress radiographs during the treatment (from Giancotti et al. [3], with kind permission of American Journal of Orthodontics and Dentofacial Orthopedics)

**Fig. 7:**
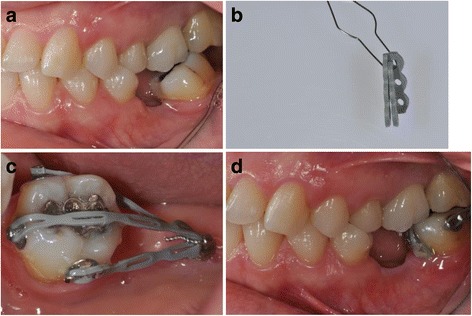
Uprighting of a mesially inclined #37 with a miniscrew implant and a G-chain. **a** Patient with missing lower first molar and mesially inclined lower left second molar before treatment. **b** A ligature wire connecting the segments of the elastic chain. **c** G-chain tied to miniscrew with ligature wire and activated by attaching free ends of chain segments to bonded buttons. **d** Clinical situation after 5 months of traction (from Greco et al. [25], with kind permission of Journal of Clinical Orthodontics)

**Fig. 8:**
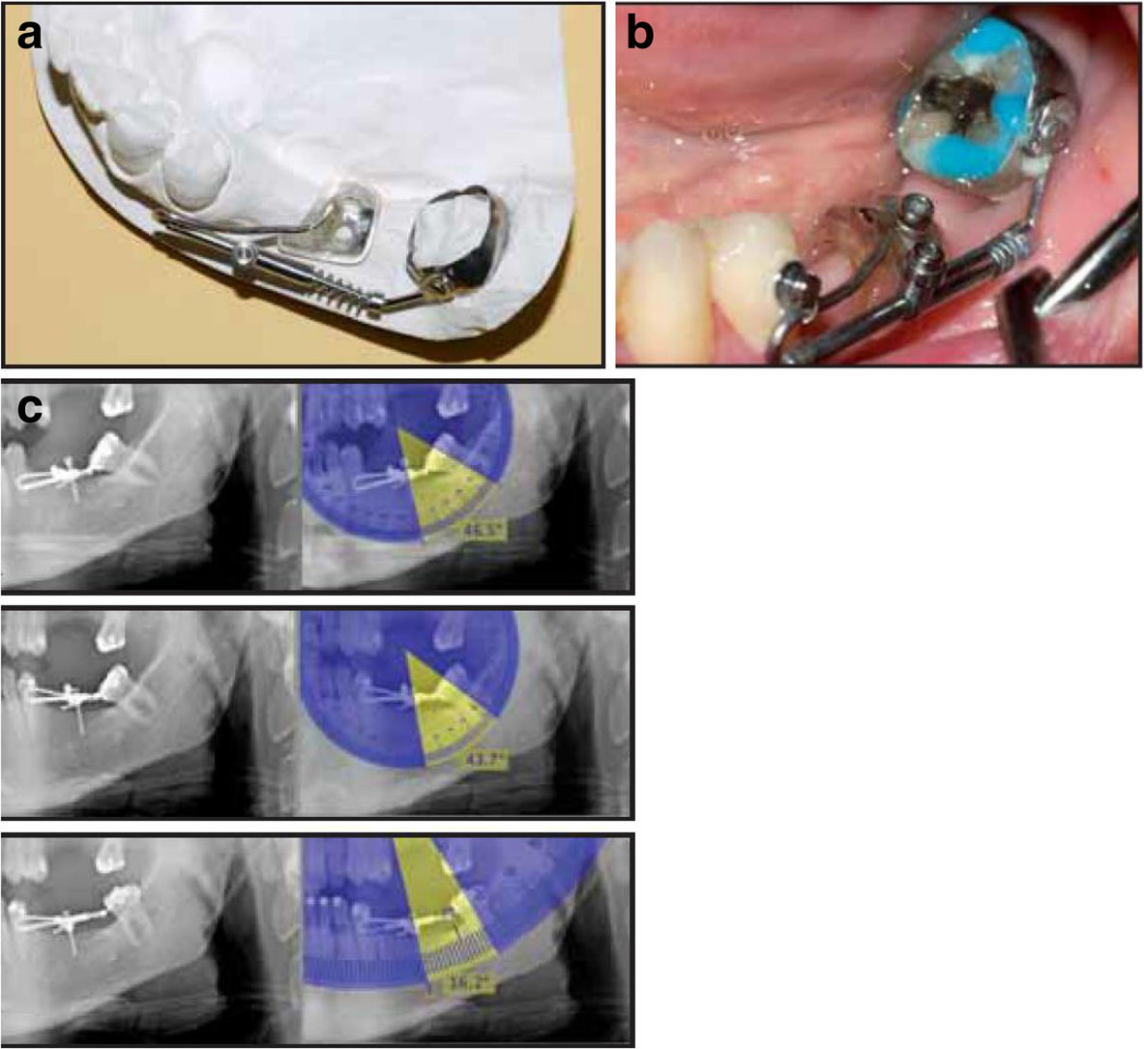
Uprighting of mesially inclined #37 with a modified Uprighter Jet. **a** Uprighter Screw consists of a molar band with a welded lingual button that allows crown rotation when a force is applied; a .036 in. tube positioned parallel to the occlusal plane; a wire with a bayonet bend on the mesial end, curving back distally from the tube, and a loop on the distal end that is screwed to the molar band; an adjustable screw clamp; and a 150-g nickel titanium open coil spring. **b** After 2 months of uprighting. **c** Radiographs with Uprighter Screw in place. **a** At the time of appliance insertion, showing 46.5° inclination of the second molarʼs long axis relative to miniscrew. **b** After 2 months of uprighting, showing 43.7° inclination. **c** After 5 months of uprighting, showing 16.2° inclination (from Gracco et al. [1], with kind permission of Journal of Clinical Orthodontics)

The origin of force differed also, as in several cases uprighting was achieved (a) by using an open or closed coil spring [3, 4] (Fig 6) or (b) by using buttons and elastomeric chains [2, 4, 5, 25, 27]. In a case of a mesially tilted second molar, buttons were placed on its labial, lingual, and mesial surface, and uprighting force was applied through three elastic chains, which were connected to a MI in the retromolar area [25]. A distalizing and uprighting movement was produced, which avoided undesirable rotation of the molar [25] (Fig 7). Other treatment options included uprighting springs [26], a small cantilever [2], a sequence of archwires and a running loop [15], and a modified version of the Uprighter Jet called Uprighter Screw [1] (Fig 8).

**Fig. 9:**
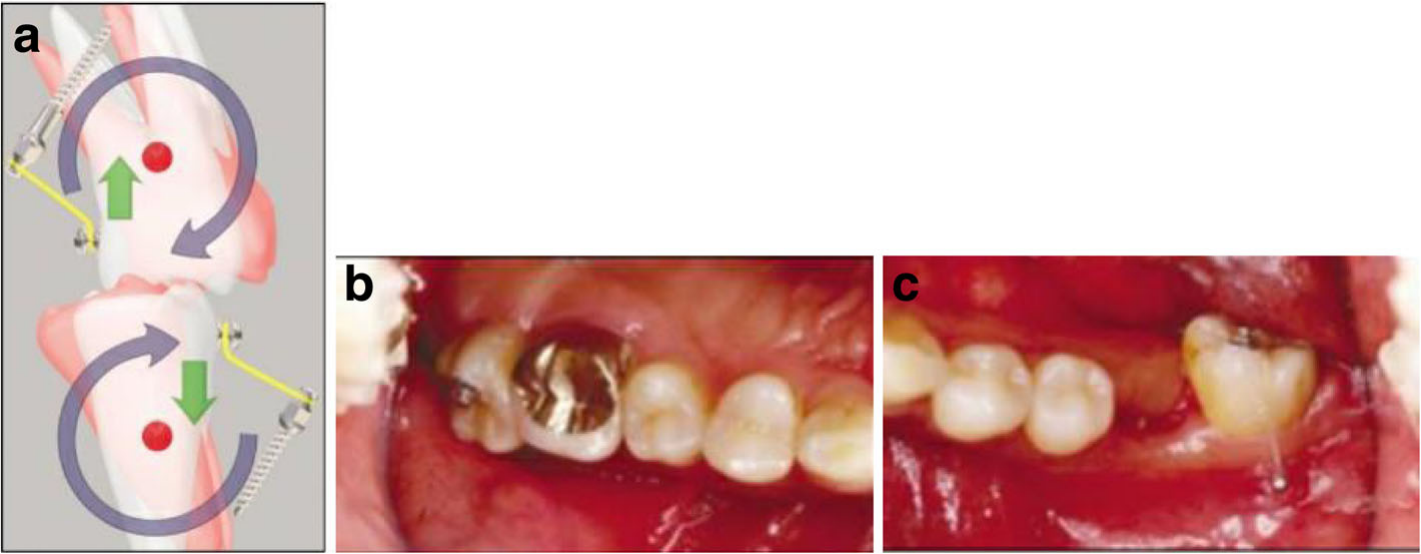
Uprighting #27 and #37 with miniscrew implants, elastomeric threads, and a temporary bite plane. **a** Force system used for molar uprighting with mini implants. **b** Elastomeric thread which was attached from mini implants to buttons bonded to occlusal surfaces of left upper **c** lower second molar (from Park et al. [28], with kind permission of Journal of Clinical Orthodontics)

As regards the direct force application on the MIs for the correction of molars on the *transverse plane*, two cases were reported [10, 28], where there was a lingually tilted second molar and a lingual crossbite of a second mandibular molar, respectively. In the first case, described by Sohn, the MI was inserted in the buccal alveolar bone and the force was applied through an elastomeric chain to a buccal button for 3 months [10]. In the second case, two MIs were placed in both the mandible and the maxilla in order to treat the crossbite with elastomeric threads attached to buttons, by producing a buccal and intrusive force on the lower molar and a palatal and intrusive force on the upper one, respectively [28] (Fig 9).

**Fig. 10:**
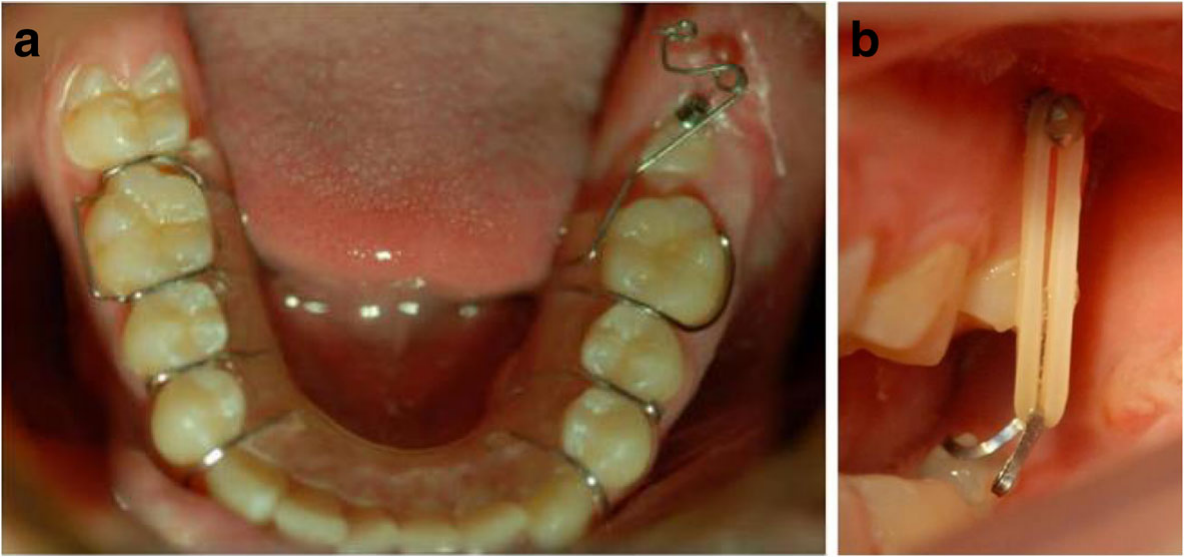
Uprighting of a mesially tipped #37 with miniscrew implants, a modified removable appliance with an uprighting spring, and an elastomeric power chain. **a** A removable appliance with an incorporated uprighting spring (0.8 mm of SS wire) that provoked the distal uprighting of the #37. **b** The buccal mini screw was inserted in the maxilla in order to initiate buccal and vertical uprighting of the lower left second molar (from Celebi et al. [29], with kind permission of Journal of Medical Cases)

Direct force application was also used in three cases, where there was a correction of molars in more than one planes of space. In the first case report [12], a second molar was mesially and lingually tipped and was simultaneously in crossbite with the maxillary molar. In this specific case, the MI was placed distobuccally in the retromolar region, and for the following 3 months, an uprighting force was applied to a lingual button on the mesiolingual surface of the second mandibular molar, through an elastomeric thread. In the second case [29], after the distal uprighting of a mesially tipped and obliquely impacted molar via a removable appliance and an uprighting spring, a MI was inserted buccally between the roots of the maxillary molars. Force was applied via an elastic chain placed between the buccal MI and a wire on the occlusal surface of the mandibular molar in order to upright the tooth buccally and vertically (Fig 10). The third case [30] describes the simultaneous uprighting and intrusion of a lingually tipped and supraerupted lower first molar. The clinicians used elastomeric chains connecting a lingual attachment on the molar and a MI placed in the interradicular region. Buccolingual uprighting of 2.3 mm and intrusion of 1.8 mm was achieved in 45 days.

### Molar uprighting using MIs with indirect anchorage

Two out of the 17 included papers, which reported only two cases, assessed molar uprighting using MIs with indirect anchorage. As mentioned above, the paper of Musilli et al. [2] presents cases treated with both types of anchorage and will be described again in the next paragraph.

**Fig. 11:**
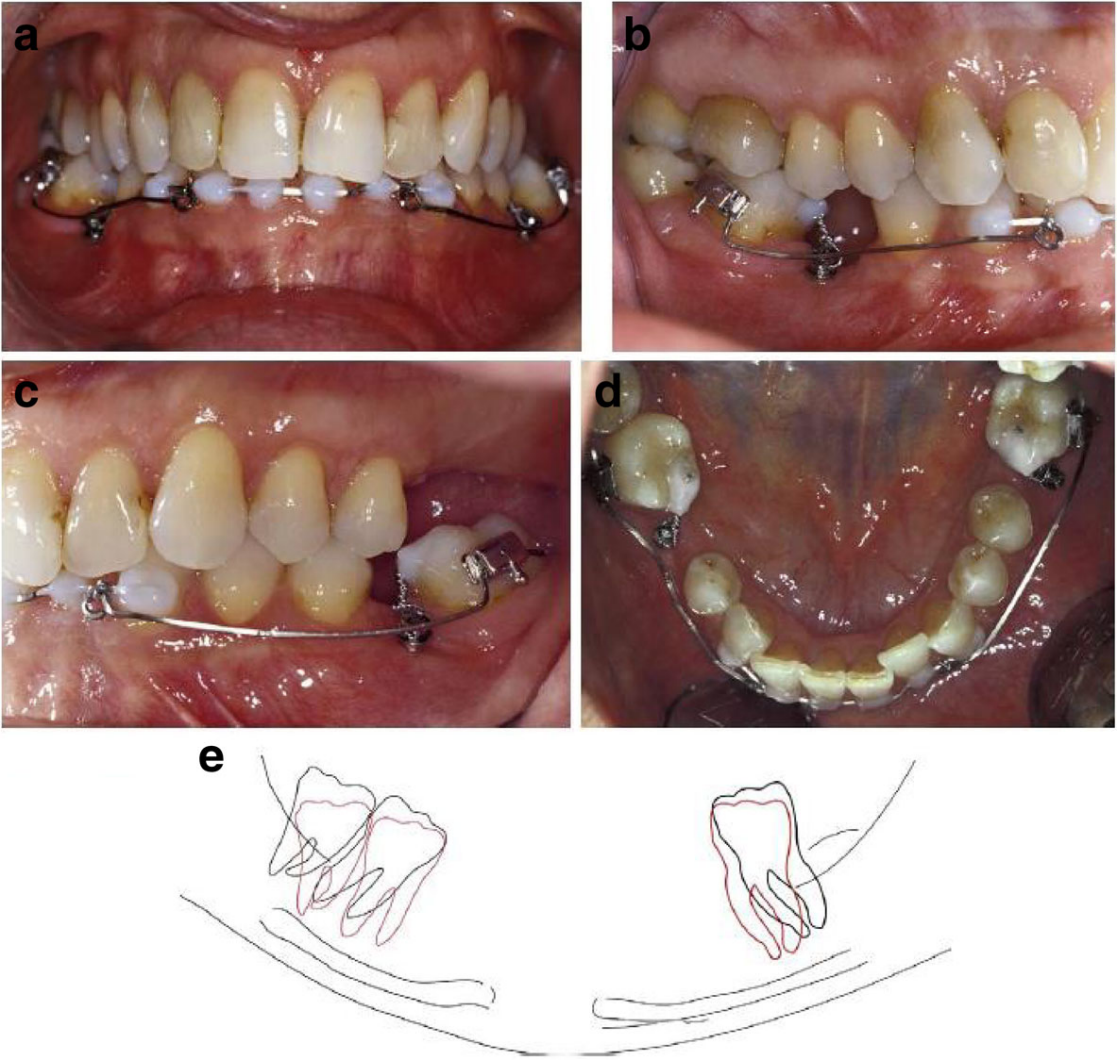
Uprighting of mesially tipped #37 and #46 with miniscrew implant and long cantilevers. The cantilevers generate molar uprighting, molar extrusion, and intrusion of the anterior teeth. Ligature between miniscrew and molar is used to control the extrusive force and the distal crown movement of each molar. Thus, the uprighting is mainly achieved by mesial root tipping. **a** The beginning of uprighting of #37 and #46 with long cantilevers, from the molar to the anterior teeth, and a screw mesial to the molar. **b** Lateral view at the initiation stage of uprighting of #46. **c** Lateral view at the initiation stage of uprighting of #37. **d** Occlusal view during the beginning stage of uprighting of #37 and #46. **e** Lateral view tracing of uprighting of #37 and #46 with superimposition. The black line stands for the initial position and the red line for the final position. The superimposition is applied on the panoramic radiograph and the mandibular canal the external oblique ridge and the lower border of the mandible are chosen as reference points (from Musilli et al. [2], with kind permission of Progress in Orthodontics)

The first case of a second mandibular molar tilted on the sagittal plane was presented by Yun et al. [13]. A MI placed between the second premolar and the first molar was connected to the tooth by a rigid stainless steel wire serving as indirect anchorage. In addition, a peerless single tube was bonded on the first molar, and a metal button was attached on the second molar, in order to achieve appropriate traction with an uprighting spring. In the second case presented by Musilli [2], two mesially tilted molars (teeth #37 and #46) were simultaneously uprighted in 9 weeks using a long cantilever on each side. Two MIs were used indirectly, locking the molars with steel ligature in order to prevent molar’s extrusion and distal tipping. Thus, the uprighting is mainly achieved by mesial root tipping (Fig 11).

### Molar uprighting using MIs both with direct and indirect anchorage

Finally, molar uprighting using MIs both with direct and indirect anchorage was the subject of the one remaining paper.

There was one clinical trial included in the current review in which 181 MIs were inserted in 102 patients and were used both as a direct and indirect anchorage for lower molar uprighting [31]. With regard to the direct anchorage, 65 MIs were inserted in 31 patients using two different options. The first option included two vertically inserted MIs in the edentulous alveolar crest area and a bracket that was bonded on a resin uniting the two MIs. A segment of wire from this bracket to the molar was activated to realize the uprighting. The second option included the use of a cantilever directly activated on a MI that was inserted perpendicular to the buccal surface of the alveolar bone. With regard to the indirect anchorage, 116 MIs were inserted in the buccal surface of the alveolar bone of 71 patients and were connected via a segment of stainless steel wire with the canine and premolars. Then, a single or a double cantilever system was used to upright the molar. According to the authors, MIs showed high success rates in both anchorage methods with a slight superiority of the direct anchorage. Only 18 MIs failed, including 15 that were used as indirect anchorage and 3 that were used as direct anchorage.

### Direct vs. indirect anchorage

According to Lee et al. [4], direct MI for molar uprighting is simpler, as it requires one MI and a single bracket or button attachment, minimizing patient’s discomfort and also reducing chair time compared to more complex indirect anchorage. Furthermore, direct MI connection with the target tooth eliminates the possibility of unwanted movement of the anchorage unit, which can occur even with indirect MI anchorage as a result of technical errors. However, direct anchorage has some limitations and especially in cases of lingually tipped or rotated molars because a single force may be insufficient to upright the tooth. Usually, such cases require a sequential application of different force systems and repeated changes of appliances. Lee et al. also report that direct MI application is not indicated in cases of extruded molars since the force system lacks an intrusive component [4].

## Discussion

This systematic review presents evidence from 17 studies that included a total of 27 cases of mandibular molar uprighting. From these 17 studies, 16 were case reports/series and only 1 was a clinical trial.

A common point of these studies is that mandibular molar uprighting is a frequent and complicated procedure, which requires good anchorage control. Even a small amount of anchorage loss can result in adverse effects on other tooth units, extrusion of the molar, or a compromised outcome. The introduction of MIs as orthodontic anchorage auxiliaries provided orthodontists with a very significant tool that will help them among others to upright molars easier, faster, with less side-effects, and less inconvenience for the patient. Moreover, in patients with many missing teeth or with periodontal compromised teeth, when conventional full-arch anchorage cannot be applied, MIs provide a unique treatment alternative to molar uprighting.

The present review incorporated an innovative pilot-formed checklist in an effort to evaluate the methodology followed in each of the included case reports/series. Although the use of the aforementioned tool is not evidence-based, it seems to provide a brief yet adequate quality analysis in matters of case reports. According to the results of the corresponding checklist, most of the cases examined were judged with rather low quality.

## Conclusions

This paper presents a variety of clinical applications of MIs in mandibular molar uprighting in all three planes of space, both with direct and indirect anchorage. Due to numerous advantages, MIs seem to constitute a reliable solution for treating tipped or impacted molars. However, since the majority of the included studies were case reports/series, which were also judged with rather low quality, the outcomes of the respective studies should be interpreted with caution and probably cannot be generalized to the average patient with similar dental malocclusions.
